# American Muslims’ Anger and Sadness about In-group Social Image

**DOI:** 10.3389/fpsyg.2016.02042

**Published:** 2017-01-11

**Authors:** Patricia M. Rodriguez Mosquera, Tasmiha Khan, Arielle Selya

**Affiliations:** ^1^Department of Psychology, Wesleyan University, MiddletownCT, USA; ^2^Department of Population Health, School of Medicine and Health Sciences, University of North Dakota, Grand ForksND, USA

**Keywords:** American Muslims, gender, perceived in-group social image, stereotypes, anger, sadness

## Abstract

We present a novel study on the role of gender in perceptions of and emotions about in-group social image among American Muslims. Two hundred and five (147 females, 58 males) American Muslims completed a questionnaire on how Muslims feel in U.S. society. The study measured both stereotypical (i.e., ‘frightening,’ ‘oppressed’) as well as non-stereotypical in-group social images (i.e., ‘powerful,’ ‘honorable’). In particular, participants were asked how much they believe Muslims are seen as ‘frightening,’ ‘oppressed,’ ‘honorable,’ and ‘powerful’ in U.S. society, and how much anger and sadness they feel about the way U.S. society views Muslims. Participants believed Muslims are seen in stereotypical ways (i.e., as ‘frightening’ and ‘oppressed’) more than in non-stereotypical ways (i.e., as ‘powerful’ and ‘honorable’). Moreover, perceived in-group social image as ‘powerful’ or ‘honorable’ did not predict the intensity of felt anger or sadness. In contrast, the more participants believed Muslims are seen as ‘frightening,’ the more intense their anger and sadness. Furthermore, responses to perceived social image as ‘oppressed’ were moderated by gender. American Muslim female participants believed that Muslims are seen as ‘oppressed’ in U.S. society to a greater extent than male participants did. In addition, perceived social image as ‘oppressed’ only predicted anger for *female* participants: the more female participants believed Muslims are seen as ‘oppressed,’ the more intense their anger. This study contributes to the scarce literature on American Muslims in psychology, and shows that both anger and sadness are relevant to the study of perceived social image.

## Introduction

In recent years, there has been a steady increase in the number of Americans who hold an unfavorable opinion of Islam and Muslims. For example, a 2010 Gallup poll showed that 43% of Americans surveyed admitted to feeling at least ‘a little’ negative toward Muslims, a number that stands in stark contrast with the 18, 15, and 8% of Americans who felt the same way toward Christians, Jews, and Buddhists, respectively (Gallup Research Center, 2010). More recently, in September 2014, 50% of Americans surveyed by the Pew Research Center believed Islam is more likely to encourage violence compared to other religions (Pew Research Center, 2014). And, in the 2016 U.S. presidential election, Donald Trump made headlines when he called for the U.S. to bar all Muslims from entering the country (The New York Times. Retrieved from http://www.nytimes.com/2015/12/09/us/politics/donald-trump-muslims.html).

These negative sentiments toward American Muslims are not new, and are sustained by pervasive societal stereotypes about this religious community. In particular, the two popular stereotypes about Muslims in the U.S. portray them as ‘prone to terrorism,’ or ‘frightening,’ and as ‘oppressed’ by their religion (Park et al., 2007; Gottschalk and Greenberg, 2008; Pratt Ewing, 2008; Lee et al., 2009; Cainkar, 2011). Furthermore, these stereotypes are gendered since they have been shown to be differentially applied to, or associated with, Muslim women or men. In particular, the stereotype of being ‘frightening’ is applied more often to Muslim men whereas the stereotype of being ‘oppressed’ is more often applied to Muslim women (Gottschalk and Greenberg, 2008; Pratt Ewing, 2008; Cainkar, 2011). Although previous research has documented the gendered nature of the two stereotypes, no study has yet examined gender differences in the extent to which these stereotypes are emotionally relevant for American Muslim women and men. In particular, is the stereotype of being ‘frightening’ more emotionally relevant to American Muslim *men* than to American Muslim women? In contrast, is the stereotype of being ‘oppressed’ more emotionally relevant to American Muslim *women* than to American Muslim men?

We present a novel study on gender differences in *perceptions of* and *emotions about in-group social image* among American Muslims. This study is novel as previous research on American Muslims has mainly focused on youth identity formation (e.g., Fine and Sirin, 2007; Sirin et al., 2010); religious coping (e.g., Abu-Raija et al., 2010; Brown et al., 2010); and emotions about the 10-year anniversary of 9/11 (Rodriguez Mosquera et al., 2013). Furthermore, sadness has typically been examined in the context of grief and bereavement (Bonanno et al., 2008). Thus, the present study also contributes to emotion research by examining sadness in a new and different social context.

### Gendered Stereotypes, Perceptions of In-group Social Image

Although anti-Muslim sentiments in the U.S. existed prior to 9/11 (Shaheen, 2009; Cainkar, 2011), negative stereotypes and attitudes toward American Muslims intensified after the 9/11 attacks since American Muslims became the focus of doubts and suspicions about their U.S. loyalties and their ties to terrorism (e.g., Rowatt et al., 2005; Park et al., 2007; Lee et al., 2009; Cainkar, 2011). For example, Park et al. (2007) measured attitudes toward Muslims, Whites, and Blacks with the implicit association test (IAT) as well as a host of explicit attitudinal measures among ethnically diverse samples of American university students. Across three studies, Park and colleagues showed that participants held more negative implicit as well as explicit attitudes toward Muslims than toward Whites or Blacks. Moreover, participants were asked to report what they knew, or have heard, about Muslims. Content analysis of participants’ responses revealed that the most common response was to associate Muslims with ‘terrorism’ (for other studies on this stereotypical view of Muslims see e.g., Rowatt et al., 2005; Lee et al., 2009; Cainkar, 2011).

Importantly, however, the stereotype that portrays Muslims as ‘prone to terrorism’ seems to be more often applied to Muslim men than Muslim women. In Gottschalk and Greenberg’s (2008) analysis of American media, Muslims were disproportionally represented as men and as ‘threatening,’ ‘frightening,’ and as having ties with ‘terrorist activities’. In contrast, when Muslim women appear in American media, they are typically portrayed as ‘oppressed’ by their religion –in the sense of being ‘voiceless’ and ‘subordinated’- and veiled (Gottschalk and Greenberg, 2008). Thus, Muslim men seem to have come to represent the stereotypical view that Islam is a dangerous religion whereas the headscarf and other forms of Islamic dress have come to symbolize the perceived oppression of women within Islam. Importantly, the stereotype of Muslim women as ‘oppressed’ is not only applied to American Muslim women who observe Islamic dress, but to American Muslim women in general (see e.g., Gottschalk and Greenberg, 2008; Pratt Ewing, 2008; Cainkar, 2011).

Although previous research has shown that societal stereotypes about Muslims are gendered, to what extent are these stereotypes reflected in American Muslims’ perceptions of their in-group’s social image? American Muslims are likely to be aware of stereotypical images of their in-group given their prevalence in American media and public discourse (e.g., Gottschalk and Greenberg, 2008; Cainkar, 2011). Thus, in the present study, we expected to find gender differences in American Muslims’ perceptions of their in-group’s social image. In particular, *American Muslim male* participants should believe that Muslims are seen as ‘frightening’ to a greater extent than *American Muslim female* participants, who should believe that Muslims are seen as ‘oppressed’ to a greater extent than their male counterparts. Further, a related but separate question concerns these perceptions’ emotional consequences. Do American Muslims feel angry and sad about how they believe their in-group is seen by others? And, are there gender differences in how much perceptions of the in-group’s social image as ‘frightening’ and ‘oppressed’ predict anger and sadness among American Muslim women and men? We turn next to this question.

### Anger and Sadness about Being Seen as ‘Frightening’ and ‘Oppressed’

Individuals feel intense anger when they, or important in-groups, are viewed negatively by others (e.g., Rodriguez Mosquera et al., 2000, 2002, 2008, 2014; Iyer and Leach, 2008)^1^. Anger is an other-focused emotion because it is typically experienced in response to negative evaluations or treatment by others (Averill, 1982; Ortony et al., 1988; Solomon, 1992; Rodriguez Mosquera et al., 2002, 2008, 2014; Shields, 2002). To feel anger is to evaluate others’ evaluations or treatment as unfair, wrong, and unjustified. As a consequence, anger is an emotion that motivates individuals to actively confront the unfair evaluation or treatment (e.g., criticism; protest; Averill, 1982; van Zomeren et al., 2004; Fischer and Roseman, 2007; Iyer and Leach, 2008; Rodriguez Mosquera et al., 2008).

In contrast, sadness is an emotional response to the perceived loss of a person, relationship, or object that is important to the individual (e.g., Frijda, 1986; Lazarus, 1991; Bonanno et al., 2008). To date, sadness has been typically studied in response to the loss of a loved one (Bonanno et al., 2008). However, sadness should also be felt in response to perceived negative social image as this signals the loss of respect or esteem from others (see also Lazarus, 1991). Studies by Abu-Lughod (2000) and by Shaver et al. (1987) support this prediction. Shaver and colleagues examined cognitive prototypes of several different emotions among American student adults. Being disapproved or disliked by others – which implies negative social image- was a frequent elicitor of sadness (Shaver et al., 1987). Furthermore, Abu-Lughod’s (2000) ethnographic work among the Awlad Ali Bedouin community in North Africa showed experiences of sadness to be intimately related to the actual or imagined loss of respect from important others (e.g., family members).

Anger and sadness about the in-group’s negative social image matter because the two emotions have distinctive and important psychological and social consequences. In a field study on emotions about the 10-year anniversary of 9/11, we examined anger and sadness’ consequences among American Muslim student and non-student adults (Rodriguez Mosquera et al., 2013). In the days prior to the anniversary, anger and sadness were associated with distinctive psychological concerns. In particular, American Muslim participants felt intense sadness about the humanitarian losses caused by the 9/11 attacks and anger about the unfair treatment their in-group suffers. Moreover, anger and sadness predicted different coping responses. Compared to anger, the emotional experience of sadness is characterized by less arousal and more apathy (Bonanno et al., 2008). Consistent with these differences, more intense sadness predicted greater rumination about the attacks whereas more intense anger predicted greater religious coping, an active coping response that included seeking social support from in-group members (Rodriguez Mosquera et al., 2013).

These two coping responses have different implications for psychological well-being and social relations. Whereas rumination exacerbates psychological distress (Hatzenbuehler et al., 2009), religious coping has been shown to predict post-traumatic growth in response to unfair evaluations and treatment among American Muslims (Abu-Raija et al., 2010). Other studies on anger have also shown this emotion to predict active coping responses that benefit the in-group, for example, willingness to protest the in-group’s unfair treatment (e.g., van Zomeren et al., 2004). Thus, compared to sadness, anger is a more empowering and beneficial response to the perceived unfair evaluation or treatment of the in-group.

In the present study, we expected American Muslim participants to feel both intense anger and sadness about perceiving that their religious in-group (i.e., Muslims) is seen as ‘frightening’ and ‘oppressed’ in U.S. society. These social images imply unfair evaluations -since they are rooted in negative stereotypes- as well as loss of social esteem. Furthermore, we also expected to find gender differences in participants’ emotional response to these perceived social images. In particular, we expected a perceived in-group social image as ‘frightening’ to be more emotionally relevant for male participants. In contrast, a perceived in-group social image as ‘oppressed’ should be more emotionally relevant for female participants.

### Present Study

The present study examined gender differences in *perceptions of* and *emotions about* perceived in-group social image among American Muslims. We measured perceived in-group social image by asking American Muslim participants to rate how much they believe a series of adjectives reflect the way Muslims are seen in U.S. society. To generate the adjectives, a group of American Muslim research assistants were consulted about adjectives that are relevant and adjectives that are *not* relevant to prevalent societal stereotypes about Muslims. The adjectives ‘frightening’ and ‘oppressed’ were chosen as reflecting current societal stereotypes of Muslim men and Muslim women that portrays them as either prone to ‘terrorist activities’ or as ‘being oppressed,’ respectively (e.g., Gottschalk and Greenberg, 2008; Cainkar, 2011). The adjectives ‘honorable’ and ‘powerful’ were chosen as adjectives that are not relevant to current stereotypes about Muslims. The inclusion of both types of adjectives –i.e., relevant and not relevant to current stereotypes of the in-group- allowed us to test the differential role of gender in stereotypical vs. non-stereotypical social images. Further, we also asked participants how much anger and sadness they feel about the way Muslims are seen in U.S. society.

We only expected gender differences for the stereotypical social images ‘frightening’ and ‘oppressed.’ More specifically, we expected American Muslim female participants to believe that Muslims are seen as ‘oppressed’ to a greater extent than male participants. Accordingly, perceiving the in-group’s social image as ‘oppressed’ should be a stronger predictor of anger and sadness for female participants. In contrast, we expected American Muslim male participants to believe that Muslims are seen as ‘frightening’ to a greater extent than female participants. Thus, perceived in-group social image as ‘frightening’ should be a stronger predictor of anger and sadness for male participants.

## Materials and Methods

### Participants

Two hundred and five (147 females, 58 males) individuals completed a questionnaire on how Muslims feel in U.S. society. Participants’ average age was 27.88 years old (SD = 11.85, range: 18–65 years old). Eighty-nine participants worked in professional occupations (e.g., teachers, lawyers, etc.), 110 participants were university students, and 6 participants did not report their profession.^2^

### Procedure

Data was collected at a public national convention of American Muslims. We adopted a culturally sensitive approach to data collection. Thus, American Muslim male and female research assistants collected the data. Participants were approached individually by the research assistants and asked if they wanted to complete a short questionnaire. All participants completed the survey individually and did not receive any financial compensation for their participation. Participants completed an informed consent and were debriefed via a written debriefing form.

### Measures

Perceived in-group social image was measured by asking participants, ‘how do you think U.S. society perceives Muslims? I believe U.S. society perceives Muslims as…’ followed by the items ‘oppressed,’ ‘frightening,’ ‘honorable,’ and ‘powerful.’ The order of item presentation was counterbalanced across participants. Participants recorded their answers on 7-point scales from 1 (*not at all*) to 7 (*extremely)*.

To measure anger and sadness, participants were asked ‘how do you feel about the way U.S. society views Muslims? I feel…’ followed by a list of emotion items. We measured anger (3 items, ‘angry,’ ‘irritated,’ ‘annoyed’ alpha: 0.68) and sadness (i.e., ‘sad’). Participants rated their emotions on 7-point scales from 1 (*not at all*) to 7 (*extremely)*.^3^

### Analyses

Due to the sample size difference in gender, we first tested the homogeneity of variances between male and female participants for each dependent variable. Although there are several tests for homogeneity of variance, we used the Fligner–Killen test because it is robust against non-normality of samples. These tests failed to find any significant difference in variance between males and females (Perceived in-group social image: ‘frightening:’ χ^2^= 1.46, *df* = 1, *p* = 0.23, ‘oppressed:’ χ^2^= 0.017, *df* = 1, *p* = 0.90; ‘honorable’: χ^2^= 0.12, *df* = 1, *p* = 0.72; ‘powerful:’ χ^2^= 0.0003, *df* = 1, *p* = 0.99; Anger: χ^2^ = 0.17, *df* = 1, *p* = 0.68; Sadness: χ^2^ = 0.040, *df* = 1, *p* = 0.84).

Next, analysis of variance (ANOVA) tests were used to examine mean differences in perceived in-group social images and emotions across gender. We run two ANOVA’s for perceived in-group social image. In the first analysis, the items ‘oppressed,’ ‘frightening,’ ‘honorable,’ and ‘powerful’ were the dependent variables. In the second analysis, we created two composite scores: stereotypical social images and non-stereotypical social images. The former composite score was the average of participants’ scores on the items ‘frightening’ and ‘oppressed,’ whereas the latter composite score was the average of participants’ scores on the items ‘powerful’ and ‘honorable.’ These composite scores were entered into a repeated measures analysis of variance with type of social image as the within-subjects factor. This analysis provided a formal test of whether participants believed their in-group is seen more in stereotypical or non-stereotypical ways. For emotions, a composite measure of anger (i.e., the average of participants’ scores on ‘angry,’ ‘irritated,’ ‘annoyed’) and the item ‘sad’ were the dependent variables. Cohen’s *d* was also calculated for each gender difference to quantify the effect size.

Further, to examine the role of gender in the associations between perceived in-group social image and the emotions, we carried out regression analyses with perceived in-group social image as ‘oppressed,’ ‘frightening,’ ‘honorable,’ ‘powerful,’ participants’ gender (dummy coded as 0 = male and 1 = female), and the interactions between each perceived in-group social image (e.g., ‘frightening’) with participants’ gender as the predictors and either anger or sadness as the outcome. Predictors were centered at their group mean. **Table 1** presents the correlations between all measures. All correlations between predictors were lower than 0.36. More specific details on each regression model are provided below in Results. For each regression, we examined the distribution of residuals and confirmed that in all cases, the residuals were approximately normally distributed.

**Table 1:** Bivariate correlations between all measures.

	1	2	3	4	5	6
1 ‘Frightening’	1					
2 ‘Oppressed’	0.22^∗∗^	1				
3 ‘Honorable’	-0.21^∗∗^	0.05	1			
4 ‘Powerful’	0.10	0.07	0.35^∗∗∗^	1		
5 Anger	0.37^∗∗∗^	0.25^∗∗∗^	-0.14^∗^	0.05	1	
6 Sadness	0.17^∗^	0.20^∗∗^	0.03	0.10	0.38^∗∗∗^	1

*‘Frightening,’ ‘Oppressed,’ ‘Honorable,’ ‘Powerful’ refer to perceived in-group social images. *^∗^p* < 0.0*5, ^∗∗^p* < 0.01, *^∗∗∗^p* < 0.001*

## Results

### Gender Effects on Mean Levels of Perceived In-group Social Image, Anger, and Sadness

**Table 2** presents means, standard deviations, univariate *F-*values, and Cohen’s *d* effect sizes for the effect of participants’ gender. For perceived-in-group social image, the multivariate main effect of participants’ gender was significant, *F*(4,192) = 5.22, *p* = 0.001, η_p_^2^ = 0.10. As expected, female participants believed that Muslims are seen as ‘oppressed’ in U.S. society to a greater extent than male participants did (see **Table 2**). This gender difference was large in magnitude based on the Cohen’s *d* value of 0.83. There were no gender differences in the extent to which participants believed Muslims are seen as ‘frightening,’ ‘honorable,’ or ‘powerful’ (see **Table 2**). Further, a repeated measures analysis of variance with type of social image as the within-subjects factor revealed that participants believed their in-group is seen more in stereotypical (*M* = 5.10, *SD* = 1.32) than non-stereotypical ways (*M* = 3.51, *SD* = 1.33; Cohen’s *d* = 1.20), *F*(1,204) = 146.74, *p* < 0.001, η_p_^2^ = 0.42.

**Table 2:** Means, standard deviations, univariate *F*’s, and Cohen’s *d* effect sizes for the effect of gender on perceived in-group social image, anger, and sadness.

	Females (*n* = 147)		Males (*n* = 58)		Univariate *F*’s			
	*M*	*SD*	*M*	*SD*	*F*	*df*	*p*	Cohen’s *d*
**In-group social image**
Frightening	5.58	1.53	5.34	1.58	1.21	1, 195	0.27	0.15
Oppressed	5.07	1.68	3.64	1.76	19.77	1, 195	<0.001	0.83
Honorable	3.27	1.60	3.43	1.53	0.14	1, 195	0.70	0.10
Powerful	3.77	1.64	3.53	1.70	0.91	1, 195	0.34	0.14
**Emotions**
Anger	4.61	1.33	4.30	1.32	2.39	1, 196	0.12	0.23
Sadness	4.30	1.61	3.82	1.61	0.88	1, 196	0.35	0.30

With regards to emotions, the multivariate main effect of participants’ gender was not significant, *F*(2, 197) = 1.14, *p* = 0.32, η_p_^2^ = 0.01. Participants reported moderately intense feelings of anger and sadness about the way Muslims are seen in U.S. society (see **Table 2**).^4^

### The Role of Gender in the Associations Between Perceived In-group Social Image, Anger, and Sadness

**FIGURE 1:**
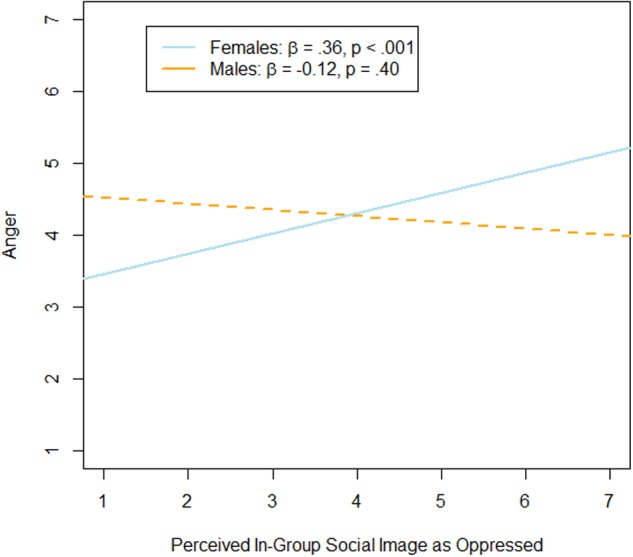
**Results of simple slope analyses.** Association between perceived in-group social image as ‘oppressed’ and anger for female and male participants.

**FIGURE 2:**
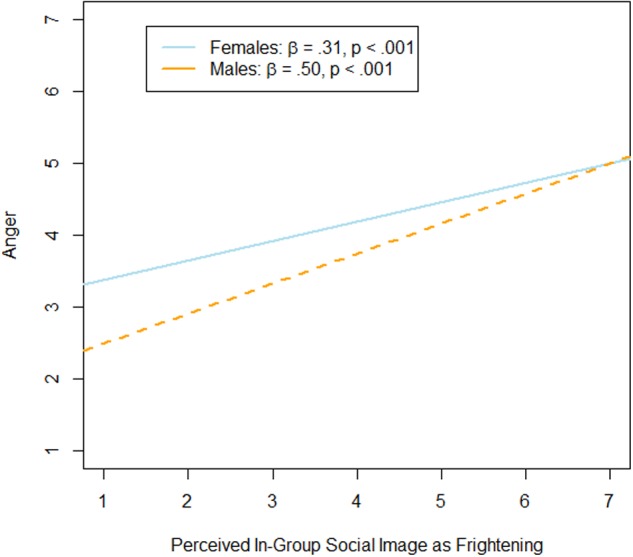
**Results of simple slope analyses.** Association between perceived in-group social image as ‘frightening’ and anger for female and male participants.

For anger, the interaction between participants’ gender and perceived in-group social image as ‘oppressed’ was significant, *b* = 0.39, *SE* = 0.11, *t* = 3.40, *p* < 0.001, 95% CI [0.16,0.62]. Simple slope analyses showed that ‘oppressed’ was a significant predictor of anger for female participants, β = 0.36, *p* < 0.001, but not for male participants, β = –0.12, *p* = 0.40 (see **Figure 1**). Further, perceived in-group social image as ‘frightening’ was also a significant predictor of anger, *b* = 0.45, *SE* = 0.11, *t* = 4.23, *p* < 0.001, 95% CI [0.24,0.65]. This main effect was qualified by a marginally significant interaction with participants’ gender, *b* = –0.24, *SE* = 0.13, *t* = –1.91, *p* = 0.058, 95% CI [–0.50,0.008]. Simple slope analyses indicated that perceived in-group social image as ‘frightening’ was a stronger predictor of anger among male participants, β = 0.50, *p* < 0.001, than among female participants, β = 0.31, *p <* 0.001 (see **Figure 2**). None of the other main or interaction effects were significant (perceived in-group social image as ‘oppressed:’ b = –0.13, SE = 0.10, *t* = –1.33, *p* = 0.18, 95% CI [–0.32,0.06], as ‘honorable:’ *b* = 0.06, *SE* = 0.11, *t* = 0.49, *p* = 0.62; 95% CI [–0.17,0.28], as ‘powerful:’ *b* = 0.002, *SE* = 0.10, *t* = 0.02, *p* = 0.99; 95% CI [–0.19,0.20]; gender: *b* = 0.25, *SE* = 0.22, *t* = 1.13, *p* = 0.26, 95% CI [–0.18,0.68]; interaction between gender and ‘honorable:’ *b* = –0.17, *SE* = 0.13, *t* = –1.27, *p* = 0.21, 95% CI [–0.43,0.09]; interaction between gender and ‘powerful:’ *b* = 0.04, *SE* = 0.12, *t* = 0.30, *p* = 0.77; 95% CI [–0.20,0.27]).

For sadness^5^, only perceived in-group social image as ‘frightening’ was a significant predictor, *b* = 0.30, *SE* = 0.14, *t* = 2.09, *p* = 0.04; 95% CI [0.02,0.58]. The more participants believed Muslims are seen as frightening in U.S. society, the more intense their sadness. None of the other main or interaction effects were significant (perceived in-group social image as ‘oppressed:’ b = 0.06, SE = 0.13, *t* = 0.42, *p* = 0.68; 95% CI [–0.20,0.32], as ‘honorable:’ *b* = 0.17, *SE* = 0.15, *t* = 1.18, *p* = 0.24; 95% CI [–0.12,0.47], as ‘powerful:’ *b* = 0.05, *SE* = 0.14, *t* = 0.34, *p* = 0.73; 95% CI [–0.22,0.32]; gender: *b* = 0.31, *SE* = 0.29, *t* = 1.05, *p* = 0.30; 95% CI [–0.27,0.89]; interaction between gender and ‘frightening:’ *b* = –0.23, *SE* = 0.17, *t* = –1.35, *p* = 0.18; 95% CI [–0.57,0.11];interaction between gender and ‘oppressed:’ *b* = 0.11, *SE* = 0.16, *t* = 0.72, *p* = 0.47; 95% CI [-0.20,0.42];interaction between gender and ‘honorable:’ *b* = –0.28, *SE* = 0.018, *t* = –1.59, *p* = 0.11; 95% CI [–0.63,0.07]; interaction between gender and ‘powerful:’ *b* = 0.06, *SE* = 0.17, *t* = 0.38, *p* = 0.71; 95% CI [–0.26,0.39]).^6, 7^

## Discussion

We presented a study on the role of gender in *perceptions of* and *emotions about* perceived in-group social image among American Muslims. We measured how much participants believed Muslims are seen in U.S. society as ‘frightening,’ ‘oppressed,’ ‘honorable,’ and ‘powerful.’ This allowed us to test the differential role of gender in stereotypical vs. non-stereotypical social images of the in-group. We also asked participants how much anger and sadness they felt about the way Muslims are seen in U.S. society. The participants believed that Muslims are seen in more stereotypical (as ‘frightening’ and ‘oppressed’) than non-stereotypical ways (as ‘honorable’ and ‘powerful’) in U.S. society. Consequently, the participants felt intense anger and sadness about how they believe their in-group is seen by others.

With regards to gender differences, we expected male participants to more strongly believe that their in-group is seen as ‘frightening’ and female participants to more strongly believe that their in-group is seen as ‘oppressed.’ In addition, we also expected a perceived in-group social image as ‘frightening’ to be a stronger predictor of male participants’ emotions and a perceived in-group social image as ‘oppressed’ to be a stronger predictor of female participants’ emotions. These expectations were based on previous research on gendered stereotypes about Muslims in the U.S. (e.g., Cainkar, 2011). Our predictions were, however, not confirmed for perceived in-group social image as ‘frightening’. In particular, both male and female participants believed to an equal extent that Muslims are seen as ‘frightening’ in U.S. society. In addition, a perceived in-group social image as ‘frightening’ predicted anger and sadness for *both* male and female participants. Thus, the more the participants believed Muslims are seen as ‘frightening’ in U.S. society, the angrier and sadder they felt.

The finding that male and female participants perceived ‘frightening’ as equally relevant for the in-group may be explained by the fact that stereotypical depictions of Muslim men are more frequent than stereotypical depictions of Muslim women in American popular media. As a consequence, Muslim men are often portrayed as representing *all Muslims* (Gottschalk and Greenberg, 2008), which could have led to female participants’ perception that this stereotype is applied to the entire in-group. It could also be the case that American Muslim women experience situations in which the stereotype is applied to them as often as American Muslim men. This personal experience with the stereotype could explain why female participants’ anger and sadness was predicted by the perception that Muslims are seen as ‘frightening.’ Future research should expand the present findings by examining the frequency with which American Muslim women and men are treated with suspicion or as a threat to others in their everyday life, and their anger and sadness about these experiences. The daily diary method is especially suitable to examine American Muslims’ everyday experiences with the stereotype.

Importantly, the participants felt *both anger and sadness* about their in-group being seen as ‘frightening.’ What are the consequences of this *joint experience* of anger and sadness about negative social image? Anger typically motivates individuals to focus on the source of the wrongdoing and to challenge the unfair treatment of the in-group by, for example, protesting or engaging in other forms of collective action (Averill, 1982; van Zomeren et al., 2004; Fischer and Roseman, 2007; Iyer and Leach, 2008; Rodriguez Mosquera, under review). In contrast, sadness is characterized by the absence of blame, an inward focus, and inaction or withdrawal (Lazarus, 1991; Bonanno et al., 2008). Furthermore, more intense sadness predicted greater rumination among American Muslims in a study on emotional responses to the 10-year anniversary of 9/11 (Rodriguez Mosquera et al., 2013). Thus, anger is motivationally a more empowering emotion than sadness is. Anger should motivate American Muslims to actively challenge the stereotypical image of their in-group as ‘frightening.’ However, sadness’ inward focus could dampen anger’s motivational benefits. Alternatively, the empowering experience of anger could lessen sadness’ negative psychological (i.e., rumination) and behavioral (i.e., inaction) consequences. Future research should examine the motivational and behavioral ramifications of this mixed emotional experience of anger and sadness about in-group social image.

Further, we found key gender differences for a perceived in-group social image as ‘oppressed.’ As expected, American Muslim *female* participants believed Muslims are seen as ‘oppressed’ to a greater extent than their male counterparts did. Moreover, perceived in-group social image as ‘oppressed’ predicted female participants’, but not male participants’, *anger*. The more female participants believed Muslims are seen as ‘oppressed,’ the more intense their anger. Interestingly, sadness was *unrelated* to ‘oppressed’ among female participants. Thus, the perception that Muslims are seen as ‘oppressed’ was uniquely tied to anger among the American Muslim women in this study. As explained above, the experience of anger is energizing and facilitates confrontational responses. The fact that it was anger, and not sadness, the emotion that was strongly associated with a perceived in-group social image as ‘oppressed’ suggests that female participants were likely in a state of ‘action readiness’ (Frijda, 1986) to confront the stereotype. In other words, they felt empowered to challenge an unfair, inaccurate image of themselves and their in-group.

In contrast to female participants’ experience, male participants saw ‘oppressed’ as less relevant to the in-group. Moreover, male participants’ emotions were *unaffected* by a perceived social image of the in-group as ‘oppressed.’ What are the consequences of these gender differences for collective action? Do these gender differences translate into American Muslim men being less willing to engage in collective action to challenge the stereotype? These questions should be examined in future research with a larger sample of American Muslim men. An important limitation of the present study was the unequal gender distribution of the sample, which was a consequence of data collection constraints.

Data collection took place at a public national convention of American Muslims. We are grateful to the organizations that allowed us to collect data for this study (please see author note) and to the conference attendees that participated in this study. Understandably, however, we were only allowed to collect data for one day and at a particular conference booth. American Muslim male and female research assistants approached attendees who walked by the booth to ask if they wanted to complete a short questionnaire. We could only take a few minutes of attendees’ time as they typically stopped by when they were going from one talk or workshop to the next. Although research assistants tried to approach an equal number of male and female attendees, many more American Muslim women completed the questionnaire. It could be the case that the conference was attended by more women than men; we were not provided with this information. Thus, we did our best to have an equal number of female and male participants in the study, but we could not achieve it given the described constraints in data collection.

Despite these limitations, the present study contributes to research on social image and emotion in important ways. In particular, the present study examined the role of gender in social image perceptions, a factor that has not been systematically examined in the social image literature (for a review, see Rodriguez Mosquera et al., 2011). In addition, sadness has mostly been studied in the context of grief and bereavement (Bonanno et al., 2008). In the present study, we examined sadness in a new and different social context. Our results show that sadness is relevant to relational losses other than the loss of a loved one. Indeed, participants felt intense sadness about the loss of social esteem implied in their in-group’s negative social image. Furthermore, the findings revealed that anger and sadness about negative social image can be concurrently felt.

Finally, the present study contributes to the scarce literature in psychology on American Muslims’ experiences. To date, research on American Muslims has examined how anti-Muslim sentiments and societal stereotypes affect American Muslims’ religious coping (e.g., Abu-Raija et al., 2010; Brown et al., 2010), youth identity formation (e.g., Fine and Sirin, 2007) and emotions in response to the 10-year anniversary of 9/11 (Rodriguez Mosquera et al., 2013). The future of psychology as an inclusive discipline lies in expanding its theories and methods to include under-studied cultural, ethnic, gender, and religious communities. Cultural psychology is key to the advancement of psychology as an inclusive discipline as it offers a variety of theoretical and methodological approaches to understand the diversity, and therefore the depth and breadth, of human experience.

## Ethics Statement

This study was approved by the Psychology Department Ethics Committee, Wesleyan University. Participants were provided with an informed consent that described the goals of the study and their rights as participants in the study. The informed consent also included contact information of the primary investigator (Dr. Rodriguez Mosquera) and the Chair of the Psychology Department at Wesleyan University in case participants wanted to send any complaints or comments about the study. Only participants who gave consent to the study were given the questionnaire. All participants received a written debriefing form.

## Author Contributions

PRM designed the study and wrote the paper. TK assisted with data collection and commented on the manuscript’s drafts. AS assisted with some statistical analyses and commented on the manuscript’s drafts.

## Conflict of Interest Statement

The authors declare that the research was conducted in the absence of any commercial or financial relationships that could be construed as a potential conflict of interest.

## Footnotes

1 The concept of perceived in-group social image relates to the concept of meta-stereotypes. Meta-stereotypes, however, are defined and typically studied in relation to a particular out-group, for example, White Canadians’ beliefs about how biased Aboriginal Canadians think they are (e.g., Vorauer et al., 1998). The focus of this paper is on American Muslims perceptions of their in-group’s social image in U.S. society, and not in relation to a particular out-group since American Muslims are a culturally and ethnically diverse group as there are, for example, African–American, Latino/a, Asian-Americans, and European–American Muslims in the U.S. (Gallup Research Center, 2011).

2 Non-student participants’ average age was 36.88 years old (*SD* = 12.61, range 19–65 years old) and student participants’ average age was 20.86 years old (*SD* = 4.07, range 18–56 years old). The average age of the six participants who did not report their occupation was 24.50 years old (*SD* = 7.50, range 18–39 years old). Because occupation and age were correlated, age was included as a covariate in all analyses of variance. We only report significant effects of age. Further, 131 participants were born in the US (97 females, 34 males) and 74 participants were born elsewhere (50 females, 24 males). Of the 74 participants who were not born in the US, 43 (58.11%) were born in Pakistan, Bangladesh, or India, 19 (25.68%) were born in a Middle Eastern country (e.g., Jordan, Iran), and the rest of the participants (16.21%) were born in Canada, the UK, Australia, the Philippines, Sudan, Nigeria, or Cuba.

3 We also measured in-group identification. As identification is a multifaceted construct (Leach et al., 2008), we measured satisfaction (e.g., “I am glad to be Muslim;” Alpha: 0.76) as this component of in-group identification is central to threat situations (Leach et al., 2008). Participants responded to the in-group satisfaction items on 7-point scales from 1 (*strongly disagree*) to 7 (*strongly agree*). Participants’ gender did not have a significant effect on in-group identification, *F*(1,199) = 0.50, *p* = 0.48, η_p_^2^ = 0.003. Participants strongly identified as Muslims (*M* = 6.75, *SD* = 0.55). Further, satisfaction was unrelated to the perceived in-group social image items, anger, or sadness (all *p’*s > 0.05).

4 Since the sample included U.S. born as well as foreign-born participants, we also included country of origin as an independent factor to explore its effects on the dependent measures. For perceived social image, the main effect of country of origin was not significant, *F*(4,192) = 1.004, *p* = 0.41, η_p_^2^ = 0.02. However, the interaction between country of origin and participants’ gender was significant, *F*(4,192) = 3.87, *p* = 0.005, η_p_^2^ = 0.07. The interaction was only significant for ‘oppressed,’ *F*(1,195) = 7.86, *p* = 0.006, η_p_^2^ = 0.04. Analyses of simple main effects revealed that *U.S. born female* participants (*M* = 5.29, *SD* = 1.66) believed Muslims are seen as ‘oppressed’ to a greater extent than non-U.S. born female participants (*M* = 4.66, *SD* = 1.66; Cohen’s *d* effect size = 0.38) and US born male participants did (*M* = 3.33, *SD* = 1.80; Cohen’s *d* effect size = 1.13), *F*(1,199) = 4.61, *p* = 0.03, η_p_^2^ = 0.02, *F*(1,199) = 33.32, *p* < 0.001, η_p_^2^ = 0.14, respectively. None of the other comparisons were significant (i.e., U.S. born and non-U.S. born males, and non-U.S. born male and females; both *p’*s > 0.05). The mean difference between U.S. born and non-U.S. born female participants is probably a consequence of the fact that the majority of immigrant female participants were born in Muslim-majority countries, where these participants were likely to be less confronted with the stereotype of Muslim women as ‘oppressed.’ Further, for emotions, country of origin had a significant multivariate effect, *F*(2,195) = 4.60, *p* = 0.01, η_p_^2^ = 0.05. In univariate terms, the effect of country of origin was only significant for anger, *F*(1,196) = 7.03, *p* = 0.009, η_p_^2^ = 0.04. U.S. born participants felt more anger (*M* = 4.74, *SD* = 1.23) about the way U.S. society views Muslims than non-U.S. born participants did (*M* = 4.14, *SD* = 1.41; Cohen’s *d* effect size = 0.45). Foreign-born participants’ less intense anger could be related to a less frequent or long-term exposure to negative stereotypes about Muslims in the U.S. (e.g., in American media) since these participants have spent less time in the U.S. compared to participants who were born in the U.S. Although the multivariate interaction between country of origin and gender was also significant, F(2,195) = 4.73, *p* = 0.01, η_p_^2^ = 0.05, none of the univariate effects were significant. Finally, the multivariate main effect of age on emotions was significant, *F*(2,195) = 4.51, *p* = 0.01, η_p_^2^ = 0.04. Age had a significant effect on sadness, *F*(1,196) = 9.03, *p* = 0.003, η_p_^2^ = 0.04. Younger participants reported more intense sadness (*r* = –0.25, *p* < 0.001).

5 The item ‘disappointed’ was originally included in the questionnaire to measure sadness. However, the correlation between sadness and disappointed was weak albeit significant: *r* = 0.25, *p* < 0.001. Given the low correlation, we did not create a composite measure of sadness by averaging the scores on the items ‘sadness’ and ‘disappointed.’ We did, however, computed regression analyses with perceived in-group social image as ‘oppressed,’ ‘frightening,’ ‘honorable,’ ‘powerful,’ participants’ gender, and the interactions between each perceived in-group social image with participants’ gender as the predictors and the item ‘disappointed’ as the outcome. Perceived in-group social image as ‘frightening’ was a significant predictor, *b* = 0.62, *SE* = 0.13, *t* = 4.86, *p* < 0.001; 95% CI [0.37,0.87]. This main effect was qualified by a significant interaction between perceived in-group social image as ‘frightening’ and participants’ gender, *b* = –0.47, *SE* = 0.15, *t* = –03.04, *p* = 0.003, 95% CI [–0.78, –0.17]. Perceived in-group social image as ‘frightening’ was a significant predictor of feeling disappointed for male participants, β = 0.47, *p* < 0.001, but not for female participants, β = 0.16, *p* = 0.06. Further, perceived in-group social image as ‘powerful’ and as ‘honorable’ were also significant predictors, *b* = –0.26, *SE* = 0.12, *t* = –2.14, *p* = 0.03; 95% CI [–0.49, –0.02], and *b* = 0.27, *SE* = 0.14, *t* = 2.02, *p* = 0.045; 95% CI [0.006,0.54]. The more participants believed Muslims are seen as ‘powerful,’ the less intense their disappointment. In contrast, the more participants believed Muslims are seen as ‘honorable,’ the more intense their disappointment. None of the other main effects or interactions were significant (perceived in-group social image as ‘oppressed:’ *b* = 0.04, SE = 0.12, *t* = 0.33, *p* = 0.74, 95% CI [–0.19,0.27]; gender: b = 0.24, SE = 0.26, *t* = 0.92, *p* = 0.36, 95% CI [–0.28,0.76]; interaction between participants’ gender and ‘oppressed:’ b = 0.12, SE = 0.14, *t* = 0.88, *p* = 0.38, 95% CI [–0.15,0.40]; interaction between participants’ gender and ‘honorable:’ b = -0.30, SE = 0.16, *t* = –1.87, *p* = 0.06, 95% CI [–0.62,0.02]; interaction between participants’ gender and ‘powerful:’ b = 0.17, SE = 0.15, *t* = 1.19, *p* = 0.23, 95% CI [–0.11,0.46]).

6 Since U.S. born participants felt more anger than non-U.S. born participants did about perceived in-group social image, we carried out additional regression analyses for anger and sadness with perceived in-group social image as ‘oppressed,’ ‘frightening,’ ‘honorable,’ ‘powerful,’ country of origin, and the interactions between country of origin and each of the social images as the predictors. Country of origin was dummy coded as 0 = born in the U.S. and 1 = not born in the U.S. For anger, only country of origin, *b* = –0.47, *SE* = 0.18, *t* = –2.60, *p* = 0.01, 95% CI [–0.83,–0.11], and ‘frightening’ were significant predictors, *b* = 0.28, *SE* = 0.08, *t* = 3.60, *p* < 0.001, 95% CI [0.13,0.44]. Having been born in the U.S. predicted greater anger. None of the other main or interaction effects were significant (perceived in-group social image as ‘oppressed:’ *b* = 0.10, *SE* = 0.06, *t* = 1.71, *p* = 0.09, 95% CI [–0.02,0.22]; as ‘honorable:’ *b* = –0.06, *SE* = 0.07, *t* = –0.80, *p* = 0.43, 95% CI [–0.20,0.08]; as ‘powerful:’ *b* = –0.02, *SE* = 0.07, *t* = –0.24, *p* = 0.81, 95% CI [–0.15,0.12]; interaction country of origin and ‘oppressed:’ *b* = 0.09, *SE* = 0.11, *t* = 0.84, *p* = 0.41, 95% CI [–0.12,0.30]; interaction country of origin and ‘frightening:’ *b* = 0.005, *SE* = 0.12, *t* = 0.04, *p* = 0.97, 95% CI [–0.24,0.25]; interaction country of origin and ‘honorable: *b* = –0.05, *SE* = 0.13, *t* = –0.40, *p* = 0.69, 95% CI [–0.31,0.21]; interaction country of origin and ‘powerful:’ *b* = 0.14, *SE* = 0.12, *t* = 1.11, *p* = 0.27, 95% CI [–0.11,0.38]). For sadness, only perceived in-group social image as ‘oppressed’ was a significant predictor, *b* = 0.17, *SE* = 0.08, *t* = 2.25, *p* = 0.03, 95% CI [0.02,0.32]. None of the other main or interaction effects were significant (‘frightening:’ *b* = 0.15, *SE* = 0.10, *t* = 1.46, *p* = 0.15, 95% CI [–0.05,0.36]; ‘honorable:’ *b* = 0.08, *SE* = 0.10, *t* = 0.81, *p* = 0.42, 95% CI [–0.11,0.27]; ‘powerful:’ *b* = 0.10, *SE* = 0.09, *t* = 1.18, *p* = 0.24, 95% CI [–0.07,0.28]; country of origin: *b* = –0.39, *SE* = 0.24, *t* = –1.65, *p* = 0.10, 95% CI [–0.86,0.08]; interaction country of origin and ‘oppressed:’ *b* = 0.03, *SE* = 0.14, *t* = –0.22, *p* = 0.83, 95% CI [–0.31,0.25]; interaction country of origin and ‘frightening:’ *b* = –0.01, *SE* = 0.16, *t* = –0.07, *p* = 0.95, 95% CI [–0.33,0.31]; interaction country of origin and ‘honorable:’ *b* = –0.28, *SE* = 0.17, *t* = –1.62, *p* = 0.11, 95% CI [–0.62,0.06]; interaction country of origin and ‘powerful:’ *b* = –0.04, *SE* = 0.16, *t* = –0.23, *p* = 0.82, 95% CI [–0.37,0.29]. Furthermore, since U.S.-born female participants scored higher on perceived in-group social image as ‘oppressed’ than non-U.S. born female participants did, we carried out additional regression analyses for anger and sadness with female participants only. In these analyses, the main effects of country of origin, perceived in-group social image as ‘oppressed,’ and their interaction were the predictors. For anger, only perceived in-group social image as ‘oppressed’ was a significant predictor, *b* = 0.28, *SE* = 0.08, *t* = 3.59, *p* < 0.001, 95% CI [0.12,0.43]; country of origin, *b* = –0.19, *SE* = 0.22, *t* = –0.85, *p* = 0.40, 95% CI [–0.63,0.25]; interaction, *b* = –0.006, *SE* = 0.13, *t* = –0.05, *p* = 0.96, 95% CI [–0.27,0.25]. For sadness, none of the main effects or the interaction were significant (‘oppressed:’ *b* = 0.17, *SE* = 0.10, *t* = 1.75, *p* = 0.08, 95% CI [–0.02,0.36]; country of origin: *b* = –0.50, *SE* = 0.28, *t* = –1.78, *p* = 0.08, 95% CI [–1.06,0.05]; interaction: *b* = –0.09, *SE* = 0.17, *t* = –0.54, *p* = 0.59, 95% CI [–0.42,0.24].

7 Finally, we carried out two additional regression analyses for anger and sadness with only the two stereotypical social images (i.e., ‘frightening,’ ‘oppressed’), participants’ gender, and the interactions between each stereotypical social image with participants’ gender as the predictors. We compared the results of these regression models with the results for the full regression models. The results did not change, with the only exception being the interaction between ‘frightening’ and participants’ gender which reached statistical significance, *b* = –0.25, *SE* = 0.12, *t* = –1.98, *p* = 0.049, 95% CI [–0.49,–0.001].
